# Comparative study of the toxicity between three non-steroidal anti-inflammatory drugs and their UV/Na_2_S_2_O_8_ degradation products on *Cyprinus carpio*

**DOI:** 10.1038/s41598-018-29524-1

**Published:** 2018-09-10

**Authors:** Xingsheng Gao, Jinju Geng, Yourong Du, Shaoli Li, Gang Wu, Yingying Fu, Hongqiang Ren

**Affiliations:** State Key Laboratory of Pollution Control and Resource Reuse, School of the Environment, Nanjing University, Jiangsu, PR of China

## Abstract

The efficiency of advanced oxidation processes (AOPs) for disposing of non-steroidal anti-inflammatory drugs (NSAIDs) has been widely studied, but the environmental fates and effects of the NSAIDs and their degradation products (DPs) are poorly understood. In this study, the efficiency of ultraviolet light/Na_2_S_2_O_8_ (UV/PS) in degrading three NSAIDs—diclofenac, naproxen, and ibuprofen—and the toxicity of their DPs on *Cyprinus carpio* (*C. carpio*) was investigated. Results showed that the three NSAIDs can be completely removed (removal rate > 99.9%) by UV/PS, while the mineralization rate of the NSAIDs was only 28%. When *C. carpio* were exposed to 0.1 μM NSAIDs, 10 μM persulfate (PS), and 0.1 μM DPs of the NSAIDs for 96 h, respectively, the toxicity effects are as the NSAID DPs > PS > NSAIDs. Research results into the time-dependent effect of NSAID DPs on *C. carpio* demonstrated that obvious toxicity effects were observed in the first 48 hours, and the toxicity effects strengthened over time. NSAID DPs may have more severe toxicity effects than NSAIDs on *C. carpio*; therefore, the operating conditions of UV/PS must be optimized to eliminate the ecotoxicity of DPs.

## Introduction

During recent decades, reports of non-steroidal anti-inflammatory drugs (NSAIDs) in lakes, streams, and groundwaters have been increasingly documented^1–3^. After adsorption to humans or animals, NSAIDs were excreted in feces or urine either as metabolites or as the unchanged parent compound, entering wastewater treatment plants (WWTPs)^4^. Diclofenac (DCF), ibuprofen (IBP), and naproxen (NPX) are the three most frequently reported NSAIDs in WWTP influents^5^, and they are ubiquitously detected in China’s main river basins, including the Pearl River^6^, Yellow River, Hai River, and Liao Rive^7^, in concentrations ranging from ng/L to μg/L. NSAIDs also are found in Europe^3,8,9^ and North America^10^.

The environmental risks posed by pharmaceuticals to aquatic organisms were assessed based on risk quotients (RQ)^11^. IBP could pose a medium risk (0.1 ≤ RQ < 1) to aquatic organisms in the Yellow River and Liao River, whereas DCF could pose a medium risk (0.1 ≤ RQ < 1) to aquatic organisms in the Hai River and a high risk (RQ ≥ 1) in the Yellow River and Liao River^7^. There is also a high risk of DCF, IBP, and NPX reported in the Danish^12^ and Norwegian^13^ aquatic environments. DCF has been added to the watch list of the “EU Water Framework Directive”^14^. Cleuvers^15^ also reflected that under field or environmental concentrations, the adverse effects caused by mixing NSAIDs can be quite toxic, even at lower concentrations.

Due to continuous and increasing NSAID consumption and their incomplete elimination in WWTPs, the presence of NSAID residues has been acknowledged as one of the most urgent emerging environmental issues, particularly in the aquatic environment^16^. WWTPs represent an obligatory and final treatment step prior to the release of NSAIDs into the aquatic media, but conventional WWTPs were not designed to remove micropollutants from wastewater. Based on the toxicological data and measurements of environmental concentrations available in literature^1,15^, the risk of acute toxic effects from these NSAIDs is unlikely, but chronic toxic effects cannot be excluded^13,17,18^. To protect water resources, reliable tertiary treatment technologies are needed to effectively remove most pharmaceuticals before secondary effluents can be discharged into the aquatic environment^19–21^.

Advanced oxidation processes (AOPs) are the most commonly used tertiary treatment in refractory pollutants degradation. Ultraviolet light/Na_2_S_2_O_8_ (UV/PS), one of the typical UV-based AOPs^22^, has attracted more interest due to the higher oxidation ability of sulfate radicals (SO_4_^−•^). The redox potential of SO_4_^−•^ is 2.6~3.1 V^23^, which is higher than that of ·OH(1.8~2.7 V), especially at neutral pH. Persulfate (PS) irradiated by UV can generate SO_4_^−•^^24^ faster than H_2_O_2_ generates·OH, due to higher molar extinction coefficients and quantum efficiency^25,26^.·OH is also generated when SO_4_^−•^ reacts with water^27^. Na_2_S_2_O_8_ is more stable and economical than H_2_O_2_^28^. During the UV/PS process, degradation products (DPs) coexist with the NSAIDs and may induce unwanted toxicity. Therefore, NSAID removal is not enough to justify the performance of UV/PS. The variation in total organic carbon (TOC) during the decomposition was measured to evaluate the mineralization of pollutants. Researchers have studied the oxidation of IBP by UV/PS^29^ and the abatement of NPX by thermally activated PS^30^, whereas the removal of TOC does not seem optimistic based on the published literature. The low TOC removal reveals that there are DPs, and these DPs may pose potential risk to aquatic organisms or human health^31^.

The environmental fates and effects of the NSAID DPs are poorly understood, although considerable persistence and bioaccumulation of NSAIDs in aquatic organisms have been reported. A suitable AOP process may reduce or eliminate the environmental effects of both pollutants and DPs; thus it is meaningful to evaluate the change in toxicity of NSAIDs and DPs during the UV/PS process. The present toxicity studies cannot reflect real environmental situations, because most acute toxicity tests are conducted at high pollutant concentrations (mg/L)—much higher than those in aquatic environments (ng/L to μg/L). Observing the long-term oxidative stress effect of aquatic organisms under environmental concentrations is more effective for evaluating the toxicity of NSAIDs and DPs.

In this study, we compared the toxicological impacts between NSAIDs and their UV/PS DPs on *Cyprinus carpio* (*C. carpio*). The *C. carpio* is a kind of native species widely distributed in China’s aquatic environment, and DCF, IBP, and NPX are the three most commonly found NSAIDs in the aquatic environment. The aim of this study is: (1) to compare the toxicity of NSAIDs and UV/PS DPs on *C. carpio*, (2) to reveal the biochemical changes when *C. carpio* is exposed to NSAID DPs by conducting dynamic experiments. This research will contribute to a better evaluation of the ecological toxicity of NSAIDs and ecological viability after UV/PS treatment.

## Results and Discussion

### Degradation of NSAIDs by UV/PS

**Figure 1:**
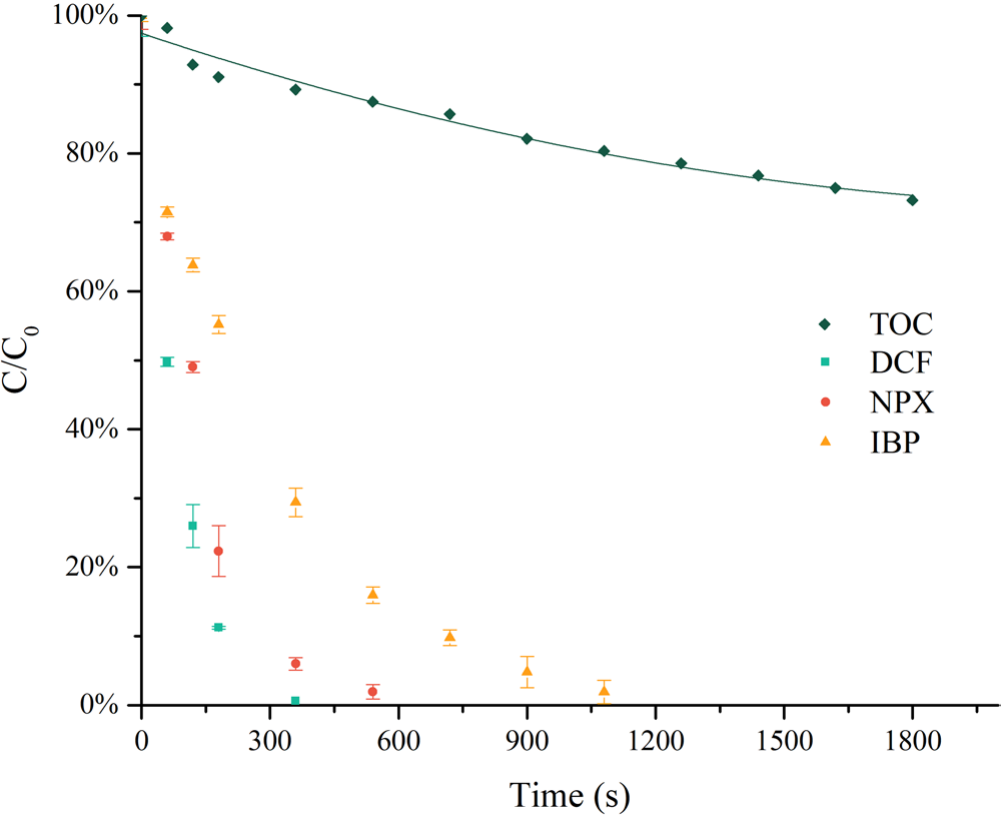
Change of the concentrations of TOC and NSAIDs during UV/PS degradation ([DCF]0 = [IBP]0 = [NPX]0 = 10 μM, [PS]0 = 1 mM).

The degradation of three NSAIDs in UV/PS and TOC changes are shown in Fig. 1. DCF was completely degraded within 6 min, and NPX in 10 min. In comparison, IBP was not fully degraded until 20 min had passed. However, only 28% TOC removal of the three NSAIDs mixtures was achieved after 30 min. The low degree of mineralization of NSAIDs and their DPs may increase the potential environmental threat of the effluents. Gao^32,33^ revealed that, in UV/PS, florfenicol was almost completely removed within 1 h, while approximately 22.3% TOC removal was achieved, and 28% of TOC removal was observed while degrading sulfamethazine. In the UV/H_2_O_2_ process, another UV-based advanced oxidation process, the TOC only decreased by 10% when sulfaquinoxaline was completely degraded^34^. Davide^35^ found that both ozonation and UV/H_2_O_2_ systems proved to be effective in DCF degradation, ensuring a complete conversion of the chlorine into chloride ions, while degrees of mineralization for ozonation was 32% and 39% for UV/H_2_O_2_ after a 90-min treatment. Similar low TOC removal results have been reported in degrading the three NSAIDs using other AOPs, such as UV/H_2_O_2_^36^, UV/O_3_^37^, and UV/TiO_2_^38^. It can be concluded that DPs cannot be mineralized effectively by AOPs, though parent compounds are largely removed. Xiang Li^39^ investigated the degradation of IBP by the electro-peroxone process, and concluded that the toxicity of the degraded IBP solution didn’t match well with the evolution of the main DPs. The electro-peroxone process can obtain a favourable TOC removal—nearly 100%—after 100 min. The evolution of the inhibition of luminescence of *Vibrio fischeri* bacteria during the electro-peroxone process rises first (from 0 to 30 min), and then descends (from 30 to 120 min). Major aromatic intermediates and phenolic intermediates detected during the electro-peroxone process reached their highest peak in 10 min, and declined to approximately 0 after 20 min. However, carboxylic acid intermediates were at high concentrations during 20~60 min.

Mass spectrum peaks of DPs formed after 5 min of UV/PS treatment are presented in Fig. S1, and Table 1 shows the hypothetical structures of DPs. Three single-component UV/PS experiments were conducted to affirm the one-to-one correspondence between DPs and each NSAID. Two structurally similar DCF DPs, DCF-DP1 and DCF-DP2, were assigned to the m/z 262 and 260, corresponding to the loss of Cl. The difference may result from the combination of two benzene rings. NPX-DP1 is characterized by the loss of H_3_CO groups and the breaking of a carbonyl group. IBP-DP1 is the main DP of IBP, generated by the decarboxylation of IBP. Michael^40^ studied the degradation of IBP under sonophotocatalysis, and also found that IBP-DP1 is the main photoproduct of IBP, with its relative intensity increasing in the first 20 min, and then decreasing. After treatment with UV/PS for 30 min, however, few DPs were detected, and the signal intensity of the DPs were lower than background value (m/z 59 of acetic acid) (Fig. S2). Under the attack of SO_4_^•−^ or other substances, hydroxylation, decarbonylation, and cyclation reaction mechanism were involved^38,40,41^. Cleavage of the aromatic rings then occurred, leading to the formation of lower molecular mass^40^. The intensity of DPs detected at 30 min were weaker than background value (m/z 59 of acetic acid), and the intensity of DPs detected at 5 min were much higher than background value. Yet the TOC removal was only 27% at 30 min and 10% at 5 min, which implied there were still many DPs that couldn’t be detected by the LTQ Orbitrap. Researchers^42,43^ have proved that residual DPs are mainly acetic acid, propionic acid, and oxalic acid. Based on the TOC results, we can conclude that the identified DPs underwent further degradation to new DPs without ionization groups that can’t be detected by electrospray ionization mass spectrometry (ESI-MS) instead of mineralization.

**Table 1:** Mass spectrometry for the identification of NSAID intermediates.

Compound	Proposed structure	molecular weight	molecular formula
DCF		296	C_14_H_11_Cl_2_NO_2_
DCF-DP1		262	C_14_H_12_ClNO_2_
DCF-DP2		260	C_14_H_10_ClNO_2_
NPX		230	C_14_H_14_O_3_
NPX-DP1		186	C_13_H_14_O
IBP		206	C_13_H_13_O_2_
IBP-DP1		161	C_12_H_17_

### Change of biochemical parameters after 96 h exposure assays

In 96 h exposure assays, the biochemical measurements revealed toxicological discrepancies on the *C. carpio* anti-oxidation system among NSAIDs, PS, and DPs.

#### Protein content

**Figure 2:**
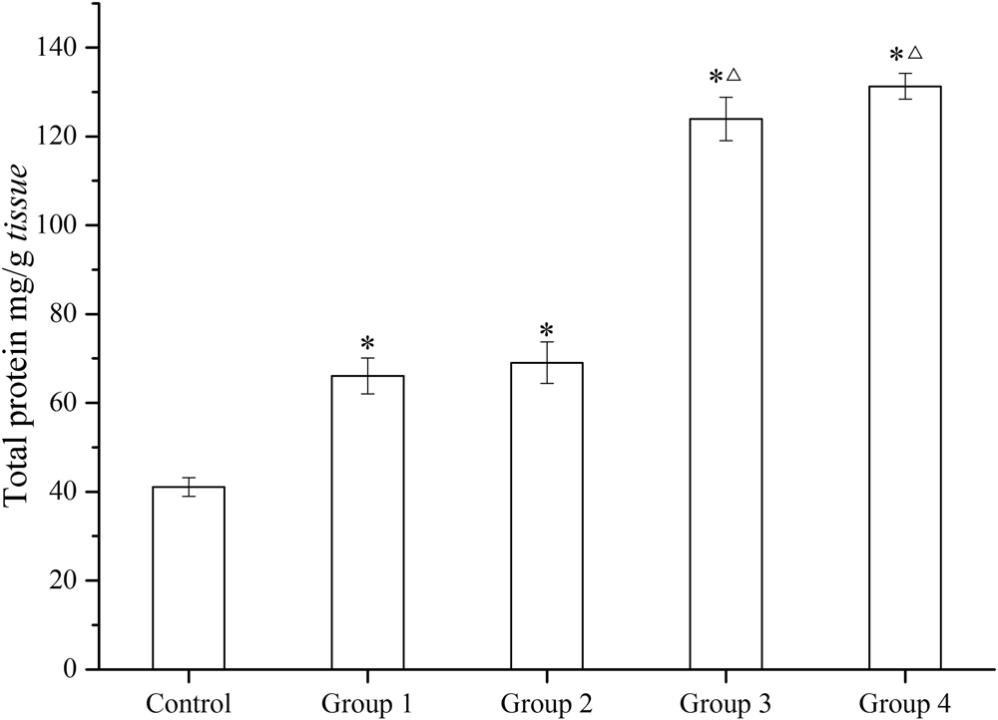
Total protein contents of *C. carpio* in 96 h exposure assays (Group 1- NSIADs, Group 2- PS, Group 3- UV/PS for 5 min, Group 4- UV/PS for 30 min). One (*) stands for significant difference (*p* < 0.05) between experiment groups and Control, and one (△) stands for significant difference (*p* < 0.05) between Group 1 and other experiment groups.

Figure 2 shows the protein content of each group. Protein content in all experimental groups increased significantly compared to the control group (Control) (*p* <0.05). Protein contents in Group 3 and Group 4 are significantly higher than that in Group 1. Protein content in Group 3 (124 mg/g) and Group 4 (131 mg/g) are 3.02 and 3.19 times of that in Control (41 mg/g), respectively, whereas protein content in Group 1 (66 mg/g) and Group 2 (69 mg/g) are 1.61 and 1.69 times of that in Control, respectively. Protein contents increased slightly along with increasing UV/PS treatment time.

#### Antioxidant enzymes

**Figure 3:**
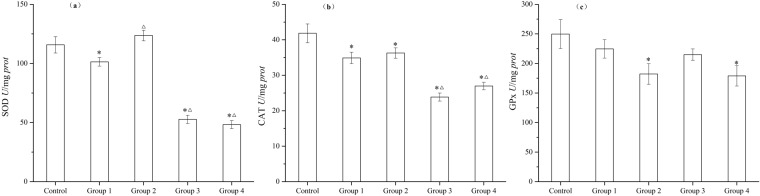
Change of antioxidant enzymes – (**a**) SOD, (**b**) CAT, and (**c**) GPx activity of *C. carpio* in 96 h exposure assays (Group 1- NSIADs, Group 2- PS, Group 3- UV/PS for 5 min, Group 4- UV/PS for 30 min). One (*) stands for significant difference (*p* < 0.05) between experiment groups and Control, and one (△) stands for significant difference (*p* < 0.05) between Group 1 and other experiment groups.

The three kinds of antioxidant enzymes such as SOD, CAT, and GPx activity counteract the adverse effects of reactive oxygen species (ROS) promoted by harmful substances accumulated in tissues^44^. Figure 3(a,b) show that the activity of both SOD and CAT clearly decreased (*p* < 0.05) in Group 3 and Group 4, with about 50% SOD inhibition and 40% CAT inhibition compared to Control, respectively. The activities of SOD and CAT in Group 3 and Group 4 were also much lower than those in Group 1 and Group 2 (*p* < 0.05). From Fig. 3(c), compared to Control, all groups showed a decrease of GPx activity, though most evidently in Group 2 and Group 4 (*p* < 0.05). Among the four groups, the more obvious inhibition of SOD, CAT, and GPx activities in Group 3 and Group 4 implied more serious oxidative damage caused by NSAID DPs. The inhibition ratio in Group 3 was SOD (54%) > CAT (43%) > GPx (14%), with Group 4 showing the same trend, SOD (58%) > CAT (36%) > GPx (29%). It can be concluded that NSAIDs treated by UV/PS for 30 min experience more severe inhibition on antioxidant enzymes compared with NSAIDs treated by UV/PS for 5 min.

#### Indicators of oxidative damage

**Figure 4:**
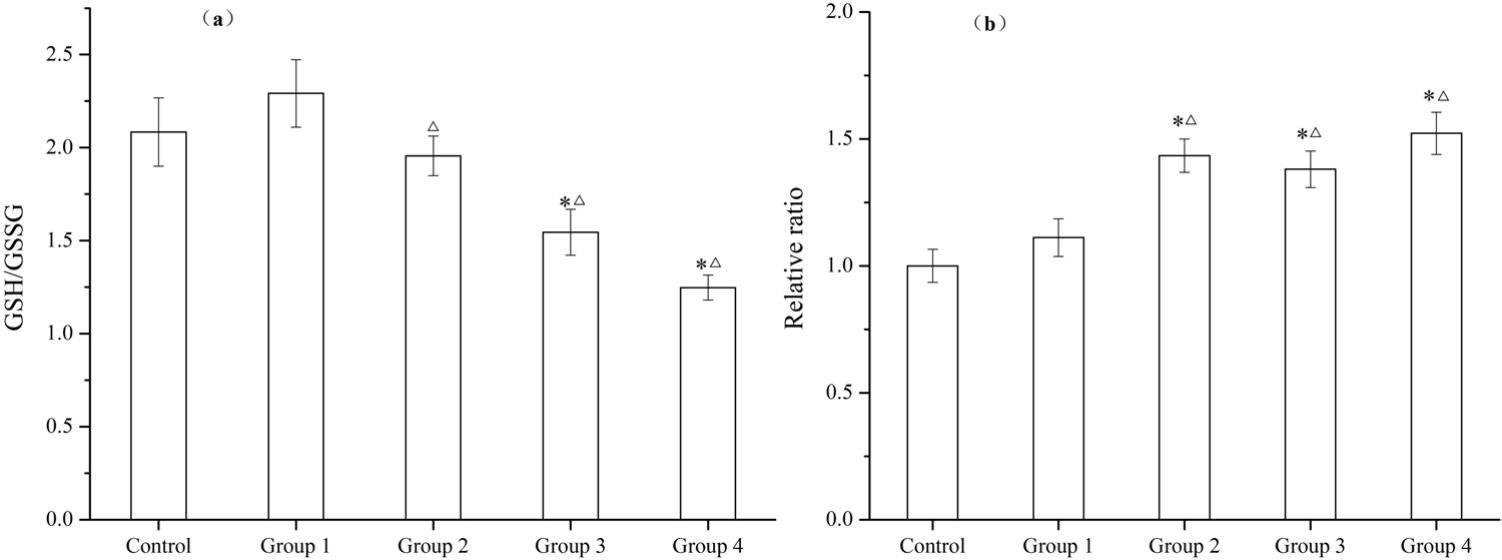
Indicators of oxidative damage – (**a**) GSH/GSSG and (**b**) MDA of *C. carpio* in 96 h exposure assays (Group 1- NSIADs, Group 2- PS, Group 3- UV/PS for 5 min, Group 4- UV/PS for 30 min). One (*) stands for significant difference (*p* < 0.05) between experiment groups and Control, and one (△) stands for significant difference (*p* < 0.05) between Group 1 and other experiment groups.

Figure 4(a) shows the GSH/GSSG ratio in *C. carpio* significantly decreased after the exposure to UV/PS-treated NSAIDs solution, with the lowest value in Group 4. No significant changes were found among Group 1, Group 2, and Control. The average MDA concentrations measured in the visceral mass from *C. carpio* are presented in Fig. 4(b). The highest concentration of MDA (5.21 nmol/mg *prot*) was obtained in Group 4, whereas the lowest concentrations (3.42 nmol/mg *prot*) was determined to be in control.

#### Discussion

When organisms live in suboptimal environments, there will be a cost to detoxification processes and other defense mechanisms in terms of metabolic resources, thus triggering increased protein synthesis^44–46^. Higher protein contents, more severe inhibition of antioxidant enzyme activities, and lower GSH/GSSG ratios revealed that DPs caused more adverse effects on fish than NSAIDs did. Studies conducted by Carregosa^47^ revealed that *Diopatra neapolitana* increased their protein content under stressful organic matter enrichment conditions. Almeida^44^ also found an increased protein content in *Scrobicularia plana* that was caused by irradiated carbamazepine.

SOD is the main enzyme responsible for offsetting the toxic effects induced by the presence of ROS, and SOD conversion of toxic superoxide anions into hydrogen peroxide is the first mechanism of antioxidation defense^48^. Afterward, CAT and GPx take part in the capture and subsequent dismutation of H_2_O_2_ to H_2_O^49^. CAT is mainly located in the peroxisomes, and is responsible for the reduction of H_2_O_2_ produced from the metabolism of long chain fatty acids in peroxisomes; GPx catalyzes the reduction of both H_2_O_2_ and lipid peroxide^50^. The different responses of CAT and GPx indicate different mechanisms for ROS removal.

Literature studies show that pharmaceuticals (especially DCF) and their photolysis byproducts were, to some extent, able to cause moderate toxicity on zebrafish after seven days of exposure^51^. Studies by Li^52^ showed the inhibition of CAT activity in the fish *Oncorhynchus mykiss* after exposure to individual carbamazepine (2 mg/L), due to the overwhelming production of hydrogen peroxide by SOD. Miguel^53^ also found that 12 μg/L cetirizine inhibited the activity of glutathione S-transferases activity (GSTs) and the activity of SOD and CAT. In this study, the obvious decrease of antioxidant enzymes activity in Group 3 and Group 4 may be due to the impairment of the antioxidant system, and responsible for the increasing lipid peroxidation and disequilibrium of GSH/GSSG.

Oxidative damage may produce DNA damage, enzymatic inactivation, and peroxidation of cell constituents, especially lipid peroxidation when antioxidant defenses are impaired or overcome^54^. The GSH/GSSG and MDA ratio in Group 1 are approximate to the levels in Control, whereas both the GSH/GSSG and MDA results indicate oxidation damage of *C. carpio* in Group 3 and Group 4 (Fig. 4). GSH is one of the most important ROS scavengers, and its ratio to GSSG can give an indication of the oxidative status of cells^44^. The normal GSH/GSSG ratio plays a key role in physiological condition; when the ratio gets lower, the antioxidant system may be impaired. The oxidative damage can also be assessed through the MDA content, which measures lipid peroxidation and is considered an indication of oxidative damage to cell membranes^55^. The results obtained reflect the consequence of UV/PS DPs having a stronger effect than PS or NSAIDs. The degree of GSH/GSSG and MDA ratio in the liver of *C. carpio* showed the toxicity order was: 30 min UV/PS treated NSAIDs (Group 4) > 5 min UV/PS treated NSAIDs (Group 3) > PS (Group 2) > NSAIDs (Group 1). According to the discrepancy between NSAIDs (Group 1) and UV/PS-treated groups (Group 3 and Group 4), we can conclude that it is the DPs that trigger cellular damage. A cytotoxicity study from Lu *et al*.^56^ showed that the DPs of DCF via UV/PS were capable of causing more toxicity than the parent compound on *Vibrio qinghaiensis*. Ferrando^57^ studied the chemotherapy drug tamoxifen by different advanced oxidation processes (UV, O_3_, UV/O_3_, UV/H_2_O_2_), and an increase of toxicity was observed during all the oxidation processes. In ozone-based treatments, the increase of toxicity was attributed to the presence of some of the DPs identified, whereas in the case of UV-based treatments there was no clear correlation between toxicity and the identified DPs.

After 96 h exposure, 0.1 μM NSAIDs, 10 μM PS and DPs of 0.1 μM NSAIDs can cause different levels of oxidative stress and oxidative damage on *C. carpio*, embodied in high protein and MDA contents, inhibited antioxidant enzymes activities and GSH/GSSG ratio. DPs of NSAIDs exhibited the most serious toxicity on *C. carpio*, followed by PS and NSAIDs. Saucedo-Vence *et al*.^58^ observed that *C. carpio* exposed to 7 mg/L DCF water was affected in oxidative stress status during the initial days of the study (at 4 days), exhibiting an increased response in blood and liver at 24 days. A higher toxicity of DPs of NSAIDs (NPX^59^, DCF^60^, IBP^37,39^) generated in UV or AOPs were observed using *Daphnia magna, Vibrio fisheri* as test organisms. According to the biochemical parameters, Group 4 (NSAIDs treated by UV/PS for 30 min) showed more severe oxidative damage than Group 3 (NSAIDs treated by UV/PS for 5 min), suggesting that more toxic DPs were produced as the UV/PS treatment time increased. Lu *et al*.^56^ found that mineralization of DCF (32% TOC removal) was limited, even with 180 min of UV/PS treatment. Additionally, the toxicity evaluation using bioluminescence of the freshwater bacterium *Vibrio qinghaiensis* suggested that more toxic degradation products and their related intermediate species formed, and the toxicity effect was temporarily reduced but not eliminated.

### Time-dependent effect of DPs of NSAIDs on *C. carpio*

The effects of NSAIDs and their transformation products that occur in the real environment and the long-term exposure effects are unknown. According to the results in 96 h exposure assays, NSAIDs treated by UV/PS showed the most serious toxicity to fish, and oxidation damage was worse in the 30-min UV/PS treatment (Group 4) than the 5-min UV/PS treatment (Group 3). To investigate the time-dependent effect of oxidative stress and oxidative damage induced by DPs of NSAIDs, we chose 5-min UV/PS-treated 0.1 μM NSAIDs as the exposure condition. There were two reasons for our choice: First, the removal of DCF and NPX was already over 90% in 5 min; second, TOC removal was only 28%, even for 30-min treatments, which implied that the DPs were reluctant to be removed by UV/PS and that a shorter treatment period could save energy.

**Figure 5:**
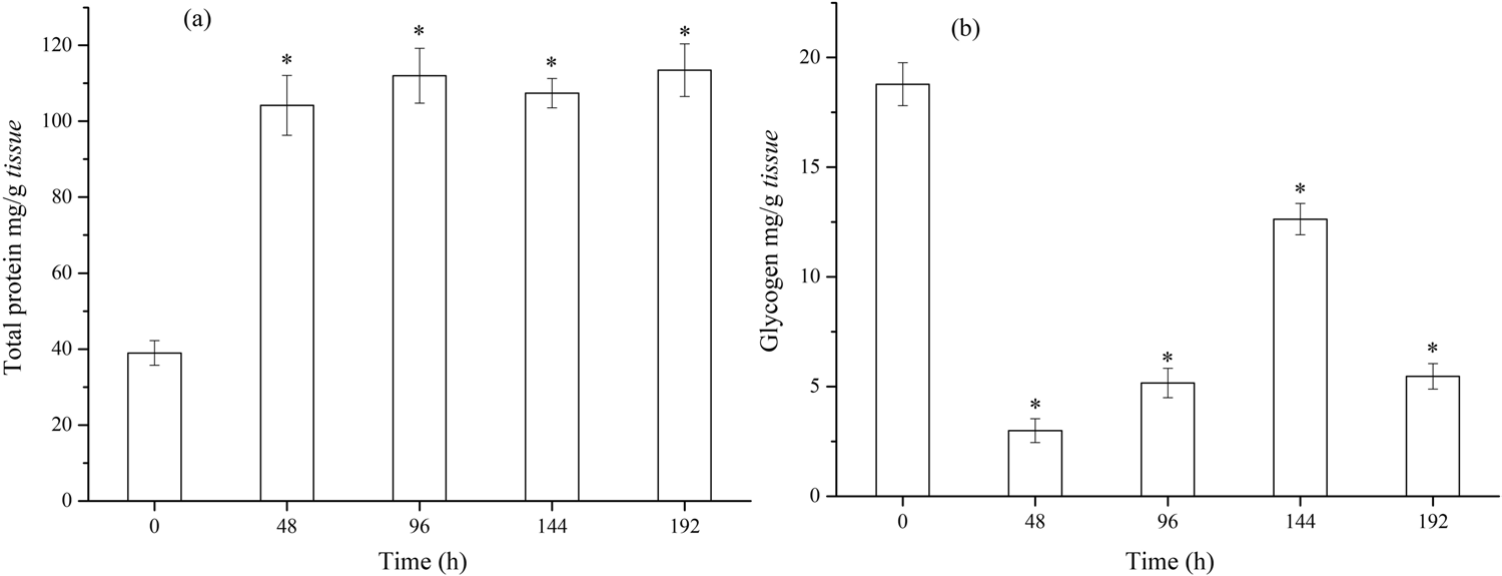
Change of energy-related parameters – (**a**) Total protein and (**b**) Glycogen of *C. carpio* in time dependent effect assays.

Figure 5(a) shows that the total protein stayed at a high level (from 104.2 to 113.5 mg/g *tissue*) during the dynamic exposure period. Figure 5(b) shows that glycogen contents dropped from 18.78 mg/g *tissue* to 2.99 mg/g *tissue* in 48 h, then rose to 12.63 mg/g *tissue* in the next 96 h, and dropped again to 5.46 mg/g *tissue* at 192 h. The glycogen content was unable to get back to the level at which it began. Protein synthesis can proceed on the condition that other sources of readily available energy, like glycogen and lipids, are sufficient^45,46^. To accommodate an adverse environment, so much glycogen was exhausted that the glycogen content could not return to its normal level through self-adjustment.

**Figure 6:**
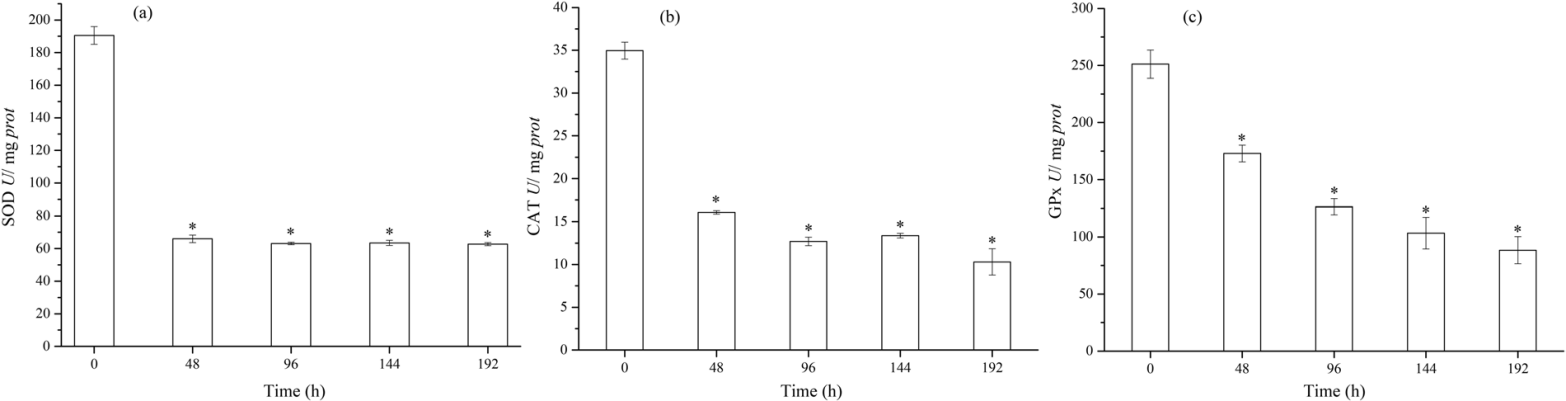
Change of antioxidant enzymes – (**a**) superoxide dismutase (SOD), catalase (CAT), and glutathione peroxidase (GPx) activity of *C. carpio* in time dependent effect assays.

**Figure 7:**
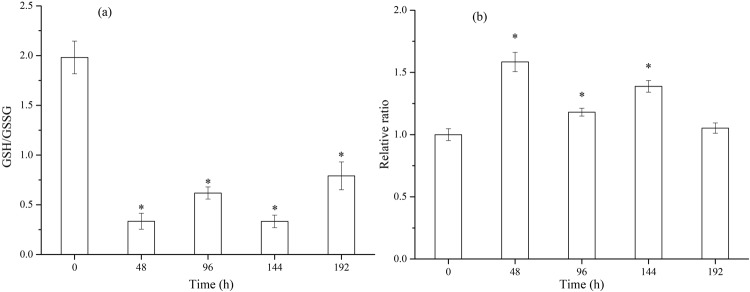
Change of indicators of oxidative stress – (**a**) GSH/GSSG ratio and (**b**) MDA ratio of *C. carpio* in time dependent effect assays.

Carbonyl groups, generated when proteins are oxidized by ROS, will decrease the catalytic activity of enzymes and increase their susceptibility to the action of proteases^61^. The activity of antioxidatant enzymes were all inhibited during the dynamic assays. Figure 6(a) shows that SOD activity was significantly inhibited (*p* < 0.01) (over 65%) from 48 h to 192 h. Figure 6(b) illustrates that an obvious decline of CAT activity was observed from 0 to 48 h (*p* < 0.01), with the activity of CAT continuing to decrease in the following 144 h. The activity of GPx, the other antioxidant enzyme, also showed a declining trend during the dynamic assays. However, it declined gradually from 0 to 192 h, in contrast to the activities of the CAT and SOD. Figure 7(a,b) show the GSH/GSSG ratio and MDA contents of *C. carpio* over different periods (0, 48, 96, 144, 192 h). When exposed to DPs of 0.1 μM NSAIDs for 48 h, the GSH/GSSG ratio declined from 1.98 to 0.33 (*p* < 0.05), and the MDA ratio reached its highest value of 1.58, significantly higher than 0 h (*p* < 0.05). From 48 h to 192 h, the ratio of GSH/GSSG fluctuated between 0.5 and 1.0, much lower than 0 h (*p* < 0.05), and MDA contents declined but remained significantly higher than 0 h, except for the value at 192 h.

In the present work, the GSH/GSSG ratio and MDA ratio of *C. carpio* reached their extreme value at 48 h, proving lipid peroxidation in the fish livers and that the fish were already experiencing oxidative damage. The low GSH/GSSG level by the end of the exposure suggested the oxidative damage occurred, although the MDA returned to a normal level. The MDA and GSH/GSSG changes in our study are similar to that of *C. carpio* exposed to pentachlorophenol^62^, glyphosate^63^, and acesulfame and its UV products^64^. Inhibited CAT and SOD activities during the exposure period further clarified that NSAID DPs caused oxidation damage in *C. carpio*. Luo *et al*.^65^ studied the toxicity of 2-chlorophenol on *C. carpio*. Seventy-two h after the intraperitoneal injection of 2-chlorophenol, the GSH/GSSG ratio returned to normal, while the MDA content stayed at a high level. Viktor^66^ studied the effects of 96 h of exposure to 7.14, 35.7, or 71.4 mg/L of Sencor (corresponding to 5, 25, or 50 mg mg/L of its herbicidal component metribuzin) on goldfish (*Carassius auratus*). Results showed Sencor exposure also induced a decrease of CAT, GPx activities, and an increase of protein content in the kidney, while the activity of SOD did not change during the experiment. Overall, the present study demonstrated 0.1 μM NSAID DPs can inflict oxidative damage on *C. carpio*. Obvious toxicity effects can be observed in the first 48 h, and the effects grow with time.

## Conclusion

UV/PS can remove DCF, IBP, and NPX effectively, but the TOC removal rate is only 28% at 30 min. Some small-molecule DPs were generated during prolonged treatment. Results from the 96 h exposure assays showed that NSAID DPs have deleterious effects on *C. carpio*, which are worse when the UV/PS treatment time is extended from 5 min to 30 min. The PS added in the UV/PS process can also trigger an oxidative stress response, but it’s not clear whether it will cause oxidative injury of the liver in a longer exposure period. NSAIDs are not as harmful to fish as PS and DPs. When *C. carpio* was exposed to NSAID DPs continuously, the antioxidant defense system sustained irreversible damage.

Our findings can be used to better evaluate the ecological safety of UV/PS in degrading the NSAIDs. Based on the research above, DPs cannot be thoroughly removed by UV/PS. Moreover, the residual DPs have more severe effects on *C. carpio*, and possibly on human health, too. Further studies are necessary to optimize UV/PS operating conditions—perhaps using a longer treatment time or higher PS dosage—to obtain better mineralization, especially for DPs, to reduce potential toxicity. The efficiency of UV/PS on wastewater also need more exploration.

## Materials and Methods

### Reagents

DCF, IBP, and NPX were purchased from Sigma-Aldrich (St. Louis, MO, USA). Methanol of HPLC-grade was supplied by Merck (Darmstadt, Germany). PS, sodium persulfate, and sodium sulfite were analytically pure and obtained from Nanjing Chemical Reagent Factory, China. Milli-Q water, with a resistivity of at least 18.2 MΩ/cm, was produced from a Millipore purification system (Billerica, CA, USA).

### UV/PS photolysis experiments

Photo degradation experiments were carried out in a photoreaction reactor (XPA-7, Nanjing Xujiang Motor Factory, China). A low-pressure mercury light (22 W, 254 nm) supplied by an electronic ballast was placed in the center of the reactor with a quartz cover. The UV lamp had an emission irradiance of 1250 μW/cm^2^ at 254 nm, as measured by a UV radiation meter (Model FZ-A, Photoelectric Instrument Factory of Beijing Normal University, China). The UV/PS photolysis experiment procedure was adjusted based on a published paper^67^. The reaction solution containing DCF, IBP, NPX (10 μM each), and PS (1 mM) was freshly prepared before the reaction experiment. In a typical reaction experiment, 50 mL of reaction solution was added into each capped quartz tube, and tubes were placed in a rotating unit at a fixed distance. A stir bar was placed inside each quartz tube to ensure that the solution was well mixed. During the photoreaction, a steady flow of cooling water was used to maintain a constant temperature of about 25 °C. A blank experiment confirmed there was no notable degradation of NSAIDs by persulfate itself during the time of the experiment.

To investigate the degradation of NSAIDs in UV/PS treatment, one quartz tube was taken out at each designated sampling time and quenched with 1 mL Na_2_SO_3_ (500 mM) for concentrations of TOC and NSAIDs detection.

For exposure assays, UV/PS photolysis experiments were carried out using the same process described above, and samples were collected at 5 or 30 min and then diluted 100 times, using de-chlorinated tap water. To avoid the influence of Na_2_SO_3_ on fish, the samples were not quenched. The diluted samples were used to refresh water in the tanks.

### *C. carpio* maintenance

*C. carpio* were purchased from a wetland park aquatic breeding base (Nanjing, China). They had a mean body length of 10.0 ± 1.0 cm, and were acclimated to laboratory conditions for two weeks before the assays. All fish were housed in three 160-L volume glass aquariums in closed circuit systems with filtered dechlorinated tap water (UV-sterilized and well-aerated) at pH 7.2 ± 0.1, a temperature of 25 ± 1 °C, and a photoperiod of 12 h light and 12 h dark as well as continuous aeration (>8 mg O_2_/L). Fish were fed commercial food once a day.

### Exposure assays

Exposure assays referred to the methods described in our previous research^64^. After being starved for 24 h, fish (*n* = 90) were gathered and then randomly distributed into 15 glass tanks. Each tank contained six fish and 25 L of test solution, with three tanks used in each treatment group. Detailed information about the exposure groups is listed in Table 2. One control group (Control) was exposed to clean tap water, and four experiment groups were exposed to a solution that was a mix of three NSAIDs (DCF, IBP, and NPX, 0.1 μM each) (Group 1), a 10 μM PS solution irradiated by UV for 30 min (Group 2), and a NSAIDs solution (DCF, IBP and NPX, 0.1 μM each) treated by UV/PS for 5 min (Group 3) or 30 min (Group 4). The concentrations of the three NSAIDs in Group 1 were all 0.1 μM—close to those in WWTP effluents^8,68^. Group 3 and 4 were set to investigate the toxicological effect of DPs as well as the time effect of UV/PS treatment. In order to reveal whether PS have adverse effect on *C. carpio* or not, Group 2 was included. During the experiment, the tanks were continuously aerated, half of the water was replaced by the same designated solution, and fish feces were removed every 24 h. At the end of the experimental period, the fish were dissected to collect their livers at 4 °C, and samples were stored at −80 °C until the biochemical parameters were analyzed.

**Table 2:** Exposure conditions of different groups.

Groups	Exposure conditions
Control	Tap water
Group 1	mixture (0.1 uM DCF + 0.1 uM IBP + 0.1 uMs NPX)
Group2	10 uM PS treated by UV for 30 min
Group3	mixture (0.1 uM DCF + 0.1 uM IBP + 0.1 uMs NPX) treated by UV/PS for 5 min
Group 4	mixture (0.1 uM DCF + 0.1 uM IBP + 0.1 uMs NPX) treated by UV/PS for 30 min

In dynamic assays, fish (*n* = 90) were exposed to NSAID DPs solution (0.1 μM NSAIDs treated by UV/PS for 5 min). The designed exposure periods were 0, 48, 96, 144, and 192 h. During the experiment, the tanks were continuously aerated, half of the water was replaced by the same designated solution, and fish feces were removed every 24 h. At the end of the experimental period, the fish were dissected to collect their livers at 4 °C, and samples were stored at −80 °C until the biochemical parameters were analyzed.

### Analytical Methods

#### Identification of NSAIDs and DPs

NSAIDs were quantified by UPLC-MS (Waters Xevo TQ-S, USA). Separation was performed using an ACQUITY UPLC BEH-C18 column (2.1 × 100 mm, 1.7 μm, Waters) at 30 °C with isocratic elution. The mobile phase consists of 10% ammonium hydroxide solution (0.01% v/v) and 90% methanol at a flow rate of 0.2 mL/min. Injection volume was 20 μL. The triple quadrupole mass spectrometer operated in electrospray negative (ESI-) mode. Data acquisition was performed by multiple reaction monitoring (MRM), recording the transitions between the precursor ion and the product ions for each target analyte. All MRM parameters were obtained by successive injections in the full chromatographic-spectrometric system, and compared with published research to ensure the reliability^69^.

The identification of NSAID DPs was performed by electrospray mass spectrometry using the LTQ Orbitrap (Thermo, USA) in negative mode. The mobile phase consists of 10% ammonium acetate solution (0.02 mM) and 90% methanol at a flow rate of 0.2 mL/min, with an injection volume of 20 μL.

#### TOC

TOC was measured using a multi N/C ®2100 TOC analyzer (Analytik Jena, German) to evaluate the mineralization of NSAIDs during the UV/PS treatment.

#### Biochemical parameters measurements

For the biomarkers measurements, liver tissue of six fish in each replicate was mixed together for one sample and then homogenized in normal saline (1:9, weight/volume). The homogenate was centrifuged for 10 min at 5000 rpm, 4 °C, and the supernatant was used for analysis. Each sample was tested in triplicate. Protein content (PROT), glycogen (GLYC), malondialdehyde content (MDA), reduced (GSH) and oxidized (GSSG) glutathione content, superoxide dismutase (SOD), catalase (CAT), and glutathione peroxidase (GPx) activity were determined using detection kits from Nanjing Jian Cheng Biology Company (Nanjing, China).

### Data analysis

Concentrations of NSAIDs and TOC were determined three times and expressed as a mean value ± standard deviation. Biochemical parameters were expressed as mean value ± standard deviation. Statistical analyses were performed using the SPSS statistical package. Variability was tested by one-way analysis of variance (ANOVA) and post Tukey HSD test using STATISTICA 22, StatSoft, Inc. (Tulsa, USA). One (*) stands for a significant difference (*p* < 0.05) between experiment groups and Control, and one (△) stands for a significant difference (*p* < 0.05) between Group 1 and other experiment groups.

### Ethical approval

All of the experimental research was performed in accordance with the National Institutes of Health Guide for the Care and Use of Laboratory Animals. The protocol was approved by the Ethics of Animal Experiments Committee of the Nanjing University.

## Electronic supplementary material

Supplementary materials
