# Author Correction: Comparative study of the toxicity between three non-steroidal anti-inflammatory drugs and their UV/Na_2_S_2_O_8_ degradation products on *Cyprinus carpio*

**DOI:** 10.1038/s41598-018-37933-5

**Published:** 2019-04-09

**Authors:** Xingsheng Gao, Jinju Geng, Yourong Du, Shaoli Li, Gang Wu, Yingying Fu, Hongqiang Ren

**Affiliations:** State Key Laboratory of Pollution Control and Resource Reuse, School of the Environment, Nanjing University, Jiangsu, PR of China

Correction to: *Scientific Reports* 10.1038/s41598-018-29524-1, published online 10 September 2018

This Article contains typographical errors in the Acknowledgements section.

“This work was supported by the National Natural Science Foundation of China (No. 21677071) and the Jiangsu Natural Science Foundation (No. BK20161474, SBK2018010218).”

should read:

“This work was supported by the National Natural Science Foundation of China (No. 21677071) and the Jiangsu Natural Science Foundation (No. BK20161474, BK20180010).”

