# Supplementary material for: Comparative study of the toxicity between three non-steroidal anti-inflammatory drugs and their UV/Na2S2O8 degradation products on Cyprinus carpio
Source: Sci Rep. 2018 Sep 10;8:13512. doi: 10.1038/s41598-018-29524-1 (PMC6131391; doi:10.1038/s41598-018-29524-1)
Supplement: Supplementary file 1 — Supplementary materials [file 41598_2018_29524_MOESM1_ESM.docx]

Comparative study of the toxicity between three non-steroidal anti-inflammatory drugs and their UV/Na_2_S_2_O_8_ degradation products on *Cyprinus carpio*

Xingsheng Gao, Jinju Geng*, Yourong Du, Shaoli Li, Gang Wu, Yingying Fu, Hongqiang Ren

State Key Laboratory of Pollution Control and Resource Reuse, School of the Environment, Nanjing University, Jiangsu, PR of China

*Corresponding author

E-mail: [jjgeng@nju.edu.cn](mailto:jjgeng@nju.edu.cn) (JG)

**Fig. S 1** Mass spectrum peak figure of three NSAIDs treated by UV/PS for 5 min

**Fig. S 2** Mass spectrum peak figure of three NSAIDs treated by UV/PS for 30 min


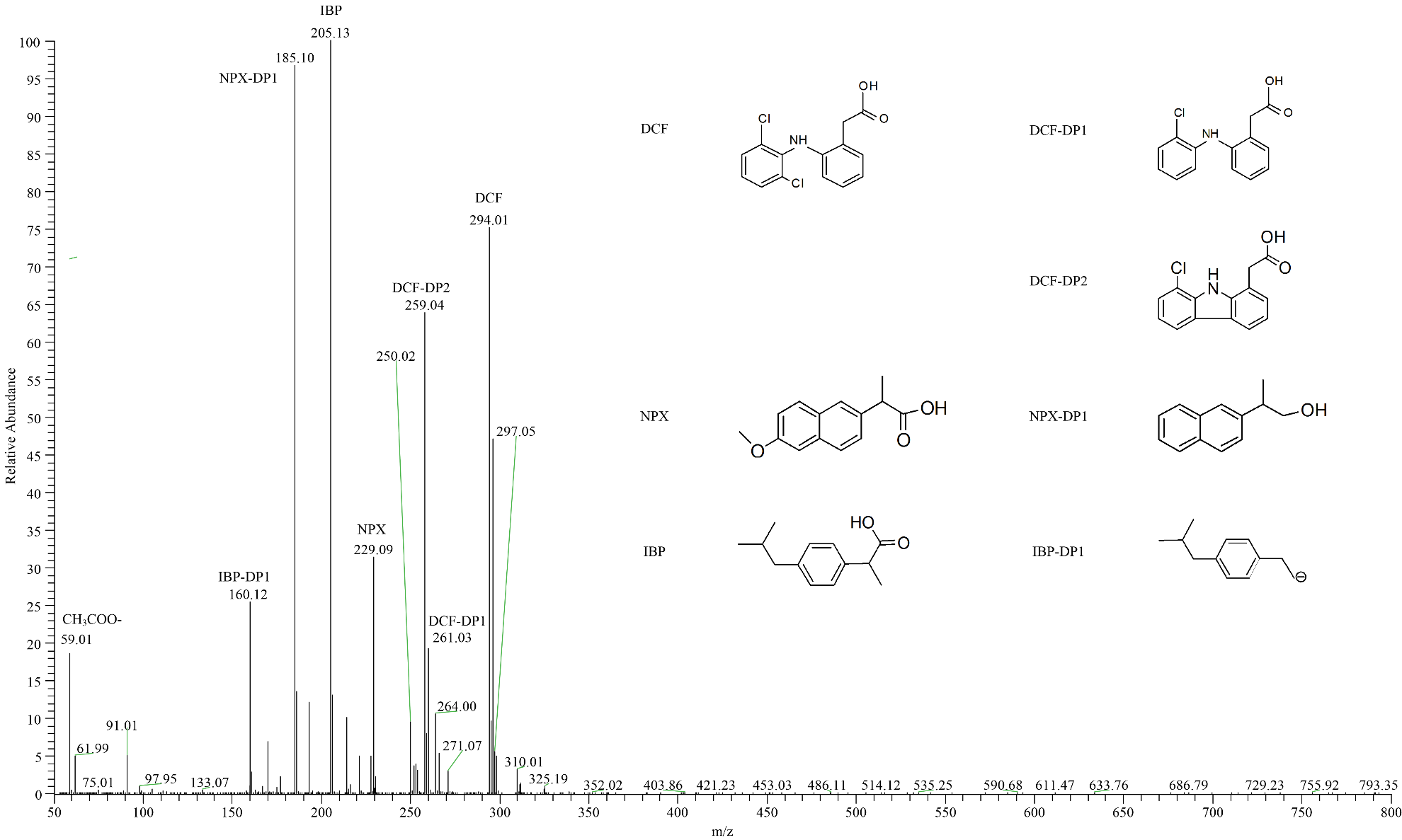


**Fig. S 1** Mass spectrum peak figure of three NSAIDs treated by UV/PS for 5 min


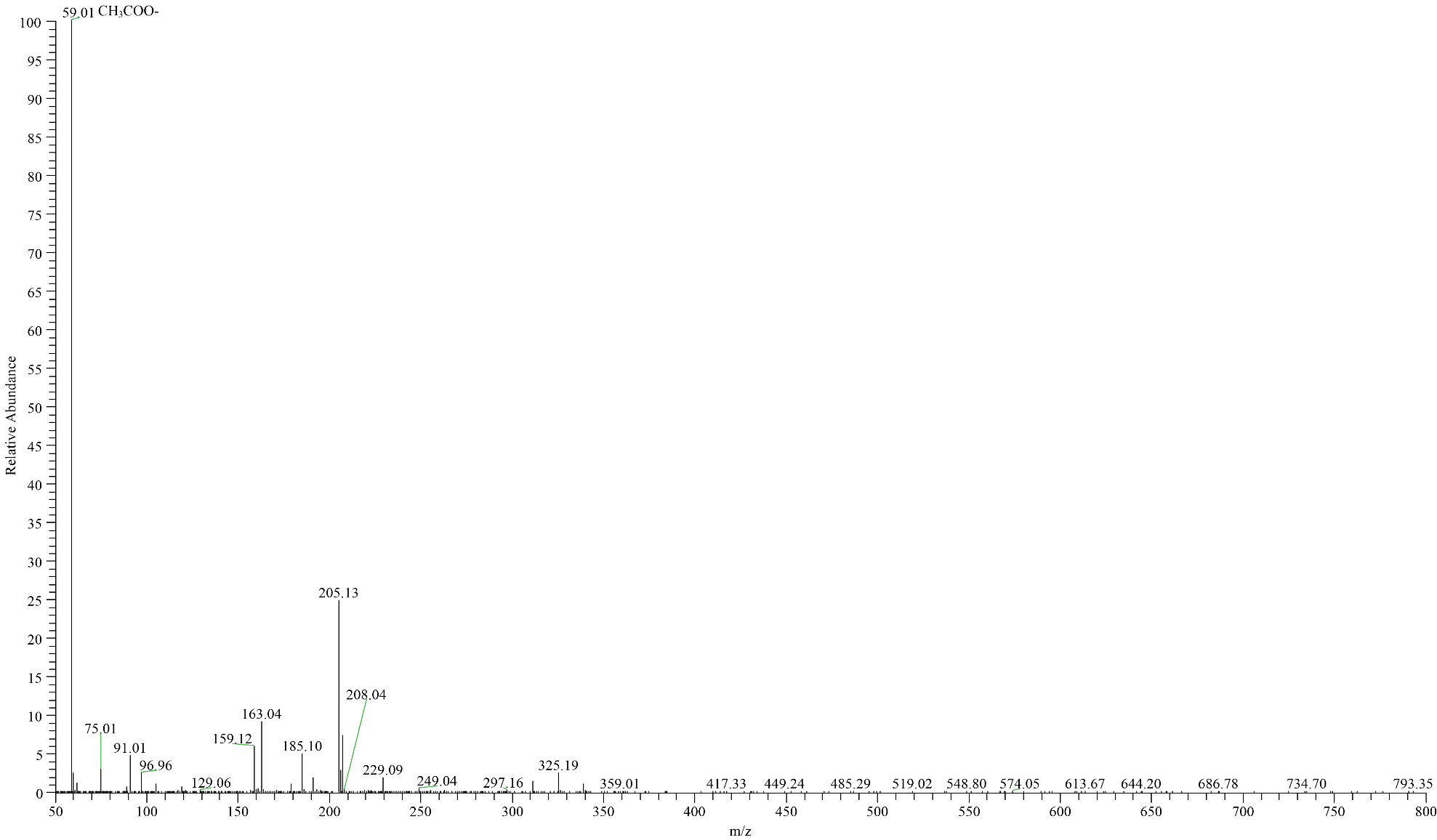


**Fig. S 2** Mass spectrum peak figure of three NSAIDs treated by UV/PS for 30 min
